# Jamming a terahertz wireless link

**DOI:** 10.1038/s41467-022-30723-8

**Published:** 2022-06-01

**Authors:** Rabi Shrestha, Hichem Guerboukha, Zhaoji Fang, Edward Knightly, Daniel M. Mittleman

**Affiliations:** 1 School of Engineering, Brown University, Providence, RI USA; 2 Department of Electrical and Computer Engineering, Rice University, Houston, TX USA

**Keywords:** Applied physics, Electrical and electronic engineering, Terahertz optics

## Abstract

As the demand for bandwidth in wireless communication increases, carrier frequencies will reach the terahertz (THz) regime. One of the common preconceived notions is that, at these high frequencies, signals can radiate with high directivity which inherently provides more secure channels. Here, we describe the first study of the vulnerability of these directional links to jamming, in which we identify several features that are distinct from the usual considerations of jamming at low frequencies. We show that the receiver’s use of an envelope detector provides the jammer with the ability to thwart active attempts to adapt to their attack. In addition, a jammer can exploit the broadband nature of typical receivers to implement a beat jamming attack, which allows them to optimize the efficacy of the interference even if their broadcast is detuned from the frequency of the intended link. Our work quantifies the increasing susceptibility of broadband receivers to jamming, revealing previously unidentified vulnerabilities which must be considered in the development of future wireless systems operating above 100 GHz.

## Abstract (web summary)

As wireless communications move towards terahertz frequencies, previously unidentified jamming vulnerabilities are revealed. Novel features not typically observed at lower frequencies are studied, including high directional attacks, beat jamming and asymmetric bit interference.

## Introduction

With the roll-out of 5G networks, consumer data consumption is expected to rise and inevitably outgrow its capabilities. Eventually, technologies beyond 5G will be necessary to fulfill future requirements. Terahertz waves (100 GHz to 10 THz) are considered a likely candidate for future generations of wireless technology^1,2^, due to the promise of large bandwidths and high data rates^3–5^. Unlike conventional radio frequency (RF) broadcasts, THz links rely on the use of highly directional beams generated from high-gain antennas designed to overcome the large free-space path loss^6,7^. This has the potential to make THz communication links more secure by restricting the ability of eavesdroppers to intercept narrow beams^8–14^. However, prior research has not addressed the security of THz links in the presence of a jammer.

At lower frequencies, jamming attacks are well known, and have been a concern since the Second World War^15^. In the simplest scenario, a jammer broadcasts in every direction in order to flood a channel and prevent the intended receiver from detecting the desired signal^16,17^. Interference and jamming are distinctly different issues that are based on the same phenomena. The former is an obstacle in establishing a proper communication link, while the latter is an attack designed with the specific goal of completely disrupting the link. The attack can dynamically vary in efficacy based on the attacker’s approach. Consequently, jamming methodologies and counter measures will necessarily evolve as the carrier frequencies increase. Because of the high directivity of THz links, the jammer will need to accurately aim at the receiver to implement a successful attack. Yet, since a primary benefit of using THz links is the large available bandwidth which promises high data rates, the receiver must have a wide operating bandwidth. This can be detrimental to the security of the link, as a jammer can take advantage of this large bandwidth for interference^18,19^. These characteristics of directivity and large bandwidths represent new considerations for jamming attacks, and therefore new challenges for system designers.

**Fig. 1:**
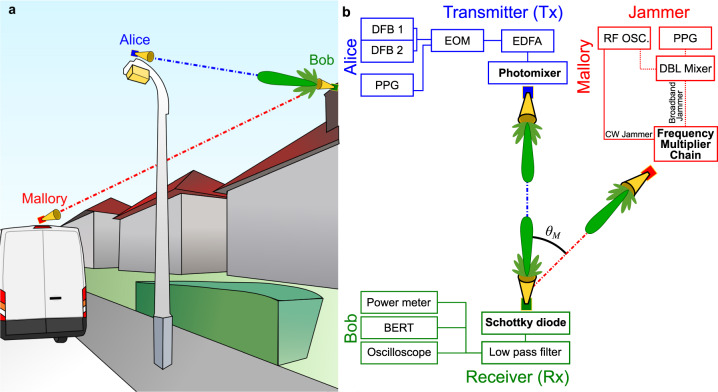
Jamming attack. **a** Alice (blue) is transmitting to Bob (green) through a line-of-sight communication link, while Mallory (red) disrupts the data streaming by aiming at Bob’s receiver, targeting one of his side lobes. **b** Schematic of the experimental setup, see “Methods” for details. BERT bit-error rate tester. DBL Mixer double balanced mixer. DFB distributed feedback laser. EOM electro-optic modulator. EDFA erbium-doped fiber amplifier. PPG pulse pattern generator. RF OSC RF Oscillator.

In this work, we perform the first study of jamming at frequencies above 100 GHz. We consider a noncoherent on–off keying modulation scheme, which is one of the two identified modes in the recent standardization of the IEEE 802.15.3d task group^20^ and is intended for simple low-cost devices. We explore several jamming scenarios that could be employed by a malicious agent to disrupt a communication link in the context of a noncoherent modulation scheme. We consider a simple threat model in which Alice (the transmitter) is communicating with Bob (the intended receiver) via a static noncoherent line-of-sight link using the main lobes of both antennas^21^. Meanwhile, Mallory (a static malicious jammer) transmits signals towards Bob at an angle *θ*_*M*_ to this line-of-sight link (see Fig. 1). We first demonstrate that a simple single-frequency tone aimed at one of the side lobes of Bob’s antenna can be effective at disrupting the intended communication, and that the effectiveness of Mallory’s attack essentially depends on her coupling to Bob’s receiver. We show that even if Bob attempts to adapt to the attack, Mallory can thwart him by varying her jamming power. More strikingly, we show that even if Mallory is not operating at the same center frequency as Alice, she can still disrupt the Alice-Bob link. This attack—which we refer to as beat jamming—reveals an important vulnerability to jamming when Alice and Bob use broad transmission bandwidths, as would be commonly expected at millimeter-wave and THz frequencies. Finally, we consider the impact on Bob when Mallory uses a modulated jamming signal, which we refer to as broadband jamming, which we found to be even more devastating as it gives Mallory additional freedom to optimize her attack.

## Results and discussion

### Single-tone jamming

We first consider the consequences for Bob if Mallory attempts to jam his signal using a single-tone frequency, which may coincide with the center frequency of Alice’s spectrum or may be at a different frequency. In our experimental configuration (see Fig. 1), Alice transmits noncoherent 1.12 Gbps on–off keying (OOK)-modulated data at a carrier frequency of 197.5 GHz. We denote the data modulation by *u*_*A*_(*t*), and her center frequency by *ν*_*A*_. Mallory is positioned off-axis at *θ*_*M*_ and attempts to disrupt the link by transmitting a single-tone frequency *ν*_*M*_ at Bob’s receiver. Bob detects signals with a zero-bias Schottky diode, which is a type of detector used for noncoherent measurement at THz frequencies and is part of the recent standardization efforts of the IEEE 802.15.3d task group^20^. As a power envelope detector, it essentially measures the square of the incident electric field *E*_*B*_, which in this case is:

1 $${|{E}_{B}(t)|}^{2}\,=	 \,|{A}_{A}{u}_{A}(t){{{{{\rm{cos }}}}}}(2\pi {\nu }_{A}t+{\varphi }_{A})\\ 	+ {A}_{M}{{{{{\rm{cos }}}}}}(2\pi {\nu }_{M}t+{\varphi }_{M})|^{2}\,+\,N(t)$$

**Fig. 2:**
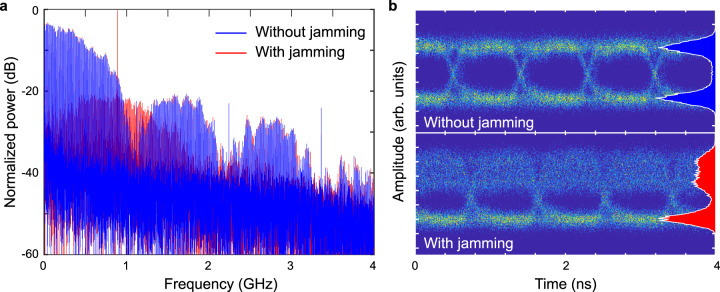
Effect of jamming. **a** Spectra and **b** eye diagrams measured by Bob, with and without the effect of jamming. In this example, the detuning Δ*ν* is 0.89 GHz, and Mallory broadcasts 10.7 dBm into Bob’s antenna side lobe, relative to Alice transmitted power of −10.5 dBm into the main antenna lobe. Mallory’s jamming introduces a wide interference component in the spectrum. In the time domain, the jamming asymmetrically affects the high bit as can be seen by comparing the histograms of data point distribution on the unjammed (top) and jammed (bottom) eye diagrams.

Here, *N*(*t*) is the noise expressed in power, and the subscripts *A*, *B*, and *M* correspond to Alice, Bob, and Mallory respectively. After factoring the expression and eliminating the high frequency and DC components—which are filtered out by Bob (see experimental setup in Methods)—the result is:

2 $${\left|{E}_{B}(t)\right|}^{2}\,=\,\frac{{A}_{A}^{2}{{u}_{A}\left(t\right)}^{2}}{2}\,+\,{A}_{A}{A}_{M}{u}_{A}\left(t\right)\,{{{{{\rm{cos }}}}}}\left(2\pi \Delta \nu t\,+\,\Delta \varphi \right)\,+\,N(t)$$

with Δ*ν* and Δ*φ* the differences in frequencies and phases between Alice and Mallory. We may assume that Δ*φ* = 0 for this analysis; this assumption does not affect any of the conclusions of this work. On the right-hand side of Eq. 2, the first term corresponds to the power of the original data stream from Alice to Bob, which is the baseband signal that is centered at 0 GHz, covering a wide spectral bandwidth determined by the data rate and modulation format (Fig. 2a). The second term is the consequence of Mallory’s jamming and consists of the product of Alice’s data stream with a sinusoidal term oscillating at the beat frequency Δ*ν*. In the spectrum, this interference term is centered at Δ*ν* with a bandwidth determined by Alice’s data rate. This is important because it means that Mallory’s jamming is never small in bandwidth compared to Alice’s; even if Mallory uses a single-frequency source, the jamming signal overlaps the spectrum of the data signal. For Bob, this means that he cannot filter out this term without also removing a significant portion of Alice’s data^22^.

The effect of Mallory’s jamming can also be observed in the time domain, through the impact on the resulting eye diagrams (Fig. 2b). In our OOK scheme, the bits are conveyed through the term *u*_*A*_(*t*), which, in its simplest form, is a non-return-to-zero rectangular function that can take values of 0 and 1. We observe that the effect of the jamming is to add noise to the high bit (the “1”), without affecting the low bit (the “0”) (Fig. 2b). This is consistent with the prediction of Eq. 2, since the jamming term is proportional to *u*_*A*_(*t*), and therefore vanishes for the zero bits. That is, when *u*_*A*_ = 0, the measured power is indistinguishable from the case without Mallory (only the noise term remains in both cases). This asymmetry between zero and one in OOK is a consequence of the use of incoherent detectors, which are often employed at these high frequencies and are explicitly incorporated as part of the approved IEEE 802.15.3d standards for the 252–325 GHz frequency range^5,20,23^. Typically, the interference model described in Eq. 2 is not observed in the vast majority of conventional low-frequency communication systems which uses coherent phase-sensitive detection. However, a similar noncoherent interference can be a characteristic of some radio technologies such as ultra-wide bandwidth (UWB) systems^24–26^.

#### Center frequency jamming

We now investigate in more detail the effectiveness of Mallory’s jamming attack when her single-tone is the same as Alice’s center frequency i.e., ${\nu }_{A}\,=\,{\nu }_{M}$. To do so, we define a metric which parameterizes this effectiveness, which we call the “jamming efficiency”, in analogy to secrecy capacity^27^:

3 $${e}_{J}\,=\,\frac{{{{{{\rm{log }}}}}}{{{{{{\rm{BER}}}}}}}_{{{{{{\rm{Unjammed}}}}}}}\,-\,{{{{{\rm{log }}}}}}{{{{{{\rm{BER}}}}}}}_{{{{{{\rm{Jammed}}}}}}}}{{{{{{\rm{log }}}}}}{{{{{{\rm{BER}}}}}}}_{{{{{{\rm{Unjammed}}}}}}}\,-\,{{{{{\rm{log }}}}}}{{{{{\rm{BE}}}}}}{{{{{{\rm{R}}}}}}}_{{{{{{\rm{Limit}}}}}}}}$$

Here, the jamming efficiency is a normalized function of the bit-error rate (BER) that Bob measures in the presence of Mallory’s jamming (${{{{{\rm{BE}}}}}}{{{{{{\rm{R}}}}}}}_{{{{{{\rm{Jammed}}}}}}}$) or in its absence (${{{{{\rm{BE}}}}}}{{{{{{\rm{R}}}}}}}_{{{{{{\rm{Unjammed}}}}}}}$). The term ${{{{{\rm{BE}}}}}}{{{{{{\rm{R}}}}}}}_{{{{{{\rm{Limit}}}}}}}$ is the BER value when Bob is completely unable to recover the signal. We consider ${{{{{\rm{BE}}}}}}{{{{{{\rm{R}}}}}}}_{{{{{{\rm{Limit}}}}}}}$ to be 10^−3^, a reasonable limit for forward error correction (FEC) algorithms^28,29^. With this definition, Mallory is ineffective with her jamming attack when *e*_*J*_ = 0, while when *e*_*J*_ ≥ 1, she has obscured the signal completely.

The success of Mallory’s attack is predicated on how efficiently she can couple her signal into Bob’s antenna relative to Alice’s coupling. This can be evaluated using the Friis transmission equation, which allows us to evaluate the ratio of powers of Alice (${P}_{{{{{{\rm{Bob}}}}}}}^{{{{{{\rm{Alice}}}}}}}$) and Mallory (${P}_{{{{{{\rm{Bob}}}}}}}^{{{{{{\rm{Mal}}}}}}}$) as measured by Bob (see Methods for the derivation):

4 $$\frac{{P}_{{{{{{\rm{Bob}}}}}}}^{{{{{{\rm{Mallory}}}}}}}}{{P}_{{{{{{\rm{Bob}}}}}}}^{{{{{{\rm{Alice}}}}}}}}\,=\,\frac{{P}_{{{{{{\rm{Mallory}}}}}}}}{{P}_{{{{{{\rm{Alice}}}}}}}}\frac{{G}_{{{{{{\rm{Mallory}}}}}}}^{\theta \,=\,0}}{{G}_{{{{{{\rm{Alice}}}}}}}^{\theta \,=\,0}}\frac{{G}_{{{{{{\rm{Bob}}}}}}}^{\theta \,=\,{\theta }_{M}}}{{G}_{{{{{{\rm{Bob}}}}}}}^{\theta \,=\,0}}{\left(\frac{{R}_{{AB}}}{{R}_{{MB}}}\right)}^{2}$$

where ${P}_{{{{{{\rm{Alice}}}}}}}$ and *P*_Mallory_ are Alice’s and Mallory’s nominal power respectively, ${G}_{{{{{{\rm{Alice}}}}}}}^{\theta \,=\,0}$ and ${G}_{{{{{{\rm{Mallory}}}}}}}^{\theta \,=\,0}$ are their respective maximal gain (in the direction of the primary lobe, *θ* = 0), and *R*_*AB*_ and *R*_*MB*_ are the distances from Alice to Bob and from Mallory to Bob respectively. Clearly, if Mallory increases her gain and/or transmit power and/or reduces her distance to Bob, she will have a more disrupting effect on the link. As for Alice, if she increases her power and/or her gain and/or reduces her distance to Bob, she can counter the effect of the jamming attack. ${G}_{{{{{{\rm{Bob}}}}}}}^{\theta \,=\,{\theta }_{M}}$ and ${G}_{{{{{{\rm{Bob}}}}}}}^{\theta \,=\,0}$ correspond to the gains of Bob’s receiver antenna in the direction of Mallory (*θ* = *θ*_*M*_) and Alice respectively. To improve the security of the link, Bob can use a more directional antenna such that the gain in the front direction is larger than the gain on the side i.e., ${G}_{{{{{{\rm{Bob}}}}}}}^{\theta \,=\,0}\,\gg\, {G}_{{Bob}}^{\theta \,=\,{\theta }_{M}}$.

**Fig. 3:**
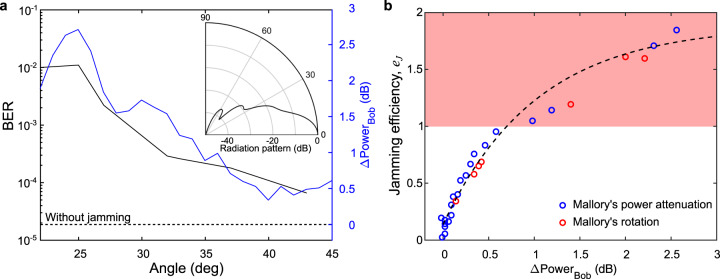
Angle of attack and jamming efficiency. **a** Mallory can reposition herself angularly to attack Bob’s side lobes (inset), which allows her to disrupt the bit-error rate (BER) and control the efficacy of her jamming. Mallory can consistently increase Bob’s BER up to 43 deg (black line) compared to if Mallory was not present (black dotted line). The inset shows the radiation pattern of Bob’s receiver and was simulated with a finite element method. **b** Alternatively, Mallory can pick an angular position respective to Bob (*θ*_*M*_ = 22°) and increase her transmit power, effectively adding power to Bob’s measurement $\Delta {{{{{\rm{Powe}}}}}}{{{{{{\rm{r}}}}}}}_{{{{{{\rm{bob}}}}}}}$, and increasing the jamming efficiency. The striped black shows an exponential fit of the data (see “Methods” for details). As Mallory’s transmit power increases, she can increase Bob’s BER up to 1.7 · 10^−3^ when transmitting a signal of 10.7 dBm, relative to Alice transmitted power of −10.5 dBm. The red region corresponds to a completely jammed signal as defined in the text following Eq. 3.

To explore these relationships, we experimentally characterize the effect of Mallory’s angular position and broadcast power on the real-time measured BER and the jamming efficiency when she uses Alice’s center frequency. Mallory directs her beam into Bob’s antenna at an angle, coupling the jamming signal into the side lobes of Bob’s antenna (20–45°). Evidently, Mallory’s jamming efficiency depends on her angular position. In Fig. 3a, we show the measured BER as a function of Mallory’s angle when she transmits a fixed power of 9.8 dBm (black curve) relative to Alice transmitted power of −10.5 dBm. For all measured angles, up to 45 degrees, Mallory’s attack degrades the BER, and induces an increase in Bob’s detected power (blue curve). Since Mallory couples into the side lobes of Bob’s antenna, it is not surprising that the BER tracks the radiation pattern (which is simulated with a finite element method in the inset of Fig. 3a). At angles of *θ*_*M*_ < 27° Mallory can access the edge of Bob’s primary lobe and increase the jamming efficiency to 100%, thus completely disrupting the link. Mallory can of course also improve the efficiency of her attack by increasing her transmitted power (and thus also increasing Bob’s received power, as noted in Eq. 4). Figure 3b shows how the jamming efficiency depends on the increase in power received by Bob, at a fixed *θ*_*M*_ = 22°. At this angle, the radiation pattern of Bob’s receiver is ~17 dB lower than the peak at 0°, which explains the required difference of nominal output power between Mallory (who is envisioned as a powerful adversary) and Alice. The results indicate that a small increase of ~0.25 dB in Bob’s received power can disrupt the link up to 50% in jamming efficiency. Moreover, if Mallory manages to increase Bob’s received power by ~0.75 dB, she can completely destroy the communication link (*e*_*J*_ > 1).

**Fig. 4:**
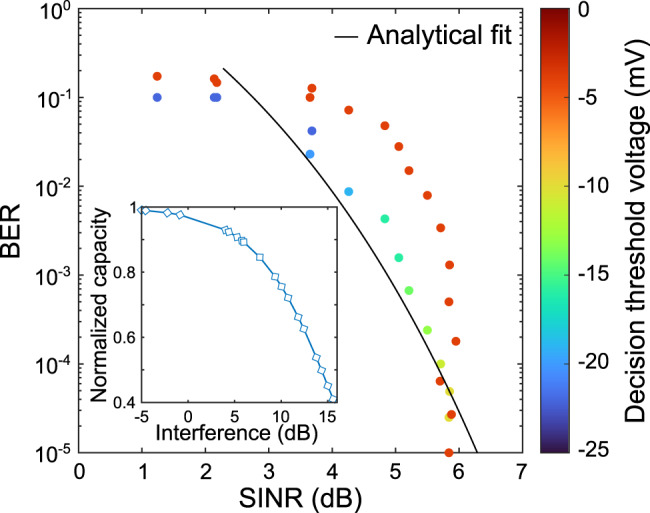
Effect of the interference and decision threshold. Experimental measurement of the bit-error rate (BER) as a function of the signal-to-interference-plus-noise ratio (SINR). The color of the dots corresponds to the value of the threshold voltage Bob uses to distinguish a one from a zero. Here, the negative value of the threshold and of the data signal is a consequence of the use of low-noise amplifiers after the Schottky diode in our particular technical configuration. The red dots are for a fixed value of the threshold initially optimized in the absence of Mallory. The results for the optimized threshold is in agreement with the general trend of the analytical expression for the error probability for noncoherent OOK system in the case of a decision point not taken at the half point (black curve, see “Methods”). The inset shows the theoretical normalized channel capacity (calculated from measurement results of the SINR) as a function of the interference contribution (see “Methods” for details).

This conclusion is not surprising and would apply equally well to jamming attacks at lower frequencies. However, the asymmetry of the effect of jamming on the 0 and 1 bits (Fig. 2b) requires a more careful consideration. The results described above assume that Bob can optimize the decision threshold used to distinguish between a 0 bit and a 1 bit, as is often the case in incoherent OOK modulation schemes. However, the asymmetry illustrated in Fig. 2b suggests that this optimization would change as Mallory varies her broadcast power, meaning that if Bob attempts to adapt to the attack, Mallory can thwart him by changing her jamming power. In Fig. 4, we compare the BER achieved by Bob with a fixed decision threshold (no optimization, red dots) to that achieved when the threshold is manually optimized at each point (yellow to blue dots). As Mallory increases her interference (decreasing the signal-to-interference-plus-noise ratio (SINR)), the BER increases in both cases. However, the variation of the BER is quite different. Remarkably, as the interference increases, Bob must monotonically decrease the value of his decision threshold to optimize the BER (as can be seen by the monotonic change of the circle colors from yellow to blue). Typically, in the presence of noise that does not distinguish between bits 0 and 1, the decision threshold is taken at the half point between the bits^30^. Here, because of the asymmetry of Mallory’s interference jamming, only the bit 1 broadens in the eye diagram (see Fig. 2b), forcing Bob to position his decision point at a lower value to correctly decipher the bits. The measured results for the case of optimized decision threshold are well described by the theoretical error probability for an incoherent OOK transmission, corrected for a decision threshold not taken at half point^30^ (black curve, see Methods). Mallory’s interference also impacts the channel capacity by effectively decreasing the SINR. The inset of Fig. 4 shows the effect of the interference on the normalized channel capacity which we obtained from experimental measurements of the SINR and the standard equation for capacity (see “Methods” for details). As the interference term increases, the normalized capacity decreases.

#### Beat jamming

**Fig. 5:**
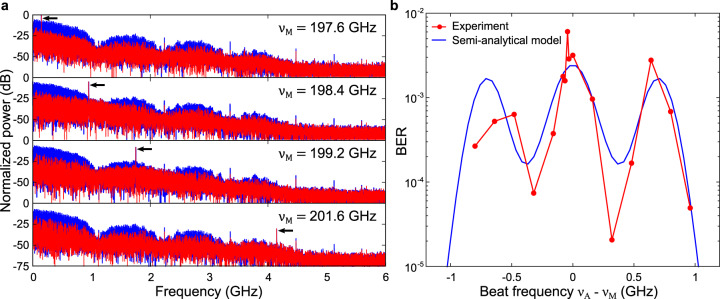
Beat jamming. **a** The received spectrum (blue) and the difference between the jammed signal and Alice’s signal (red) are shown, for four different values of Mallory’s single-tone frequency, *ν*_*M*_. As Mallory’s frequency increases, the interference signal and beat signal location (shown with the arrow) on the spectrum increase as well. **b** Measured BER (red dots) as a function of the difference in frequency between Alice and Mallory (${\nu }_{A}\,-\,{\nu }_{M}$). The observed oscillatory behavior can be modeled (blue line) using a simple description of the SINR (Eq. 5) and the BER fit of Fig. 4. Here, we used *A*_*a*_ = 1, *A*_*m*_ = 0.17 for the amplitudes, ${{{{{\rm{SINR}}}}}}\,=\,19\,{{{{{\rm{dB}}}}}}$ in the absence of interference, and $T\,=\,0.8929\,{{{{{\rm{ns}}}}}}$, which corresponds to the experimental data rate $B\,=\,1.12\,{{{{{\rm{Gbps}}}}}}$. In these measurements, Alice’s frequency is fixed at 197.5 GHz.

We next consider the possibility that Mallory’s single-tone frequency may be different from Alice’s i.e., ${\nu }_{A}\,\ne\, {\nu }_{M}$. As noted above, this possibility—which we refer to as beat jamming—has not previously been considered in the context of malicious jamming attacks, due to the narrowband nature of typical receivers at lower frequencies^16,31^. Here, because Bob’s receiver likely operates with a broad detection bandwidth, Mallory has this additional degree of freedom to optimize the effectiveness of her attack while minimizing the likelihood of alerting Alice and Bob to her presence. In our experiment, Mallory is positioned at *θ*_*M*_ = 22°, transmits 12 dB more power than Alice and sweeps her frequency from 197.6 to 201.6 GHz, while Alice’s center frequency is fixed at 197.5 GHz. In Fig. 5a, we show the effect of Mallory’s jamming on the spectrum measured by Bob, for various values of Δ*ν*. The blue curves are the jammed spectrum, and the red curves show the difference between this jammed spectrum and the original unjammed spectrum. Mallory’s jamming introduces a broad interference term spectrally centered at the beat frequency Δ*ν* (indicated by the black arrow in Fig. 5a). As Mallory’s transmit frequency increases, the position of the interference signal in Bob’s spectrum is shifted towards higher frequencies. A separate measurement when Alice and Mallory transmit single-tone signals without modulation (not shown) confirms the location of the beat frequency.

In this attack scenario, the effect of Mallory’s interference depends on the bandwidth and modulation format employed by Alice. We observe an oscillatory behavior in the BER with an increase in Mallory’s frequency (red circles in Fig. 5b). This is because, as she increases her frequency, the interference she introduces overlaps with the peaks and valleys of the spectrum of the original data, causing respectively greater and lower degradation of the communication link. We can model this phenomenon based on a simple description of the SINR. From Eq. 2, we assume that the first, second and third terms correspond respectively to the signal *S*, the interference *I* and the noise *N*. We consider a spectral representation of the individual terms, which we integrate over the bandwidth *B* corresponding to the main spectral lobe:

5 $${{{{{\rm{SINR}}}}}}\,=\,\frac{{\int }_{0}^{B}\left|\widetilde{S}\left(\nu \right)\right|d\nu }{{\int }_{0}^{B}\left|\widetilde{I}\left(\nu \right)\right|d\nu \,+\,{\int }_{0}^{B}\left|\widetilde{N}\left(\nu \right)\right|d\nu }$$

where $\left|\widetilde{S}\left(\nu \right)\right|$, $\left|\widetilde{I}\left(\nu \right)\right|$, and $\left|\widetilde{N}\left(\nu \right)\right|$ are the absolute values of the Fourier transforms of the power terms corresponding to the signal, the interference, and the noise, respectively first, second and third term of Eq. 2. Here, the frequency integrals are taken between 0 and the bandwidth *B*. In practice, this could represent the use of a carefully designed bandpass filter that could remove the out-of-band noise and interference added by Mallory’s jamming. In particular, the interference term becomes

6 $$\widetilde{I}\left(\nu \right)={\mathfrak{I}}\left\{I\left(t\right)\right\}\,=\,{A}_{A}{A}_{M}{\mathfrak{I}}\left\{{u}_{A}\left(t\right)\right\}* {\mathfrak{I}}\left\{{{{{{\rm{cos }}}}}}\left(2\pi \Delta {{\nu }}t\right)\right\}$$

where ${\mathfrak{I}}{\mathfrak{\{}}{\mathfrak{\}}}$ denotes the Fourier transform and * is the convolution operator. The Fourier transform of the cosine gives rise to two delta functions centered at the frequencies ±Δ*ν*, which, by convolution and the sifting property, shift the data spectrum ${\mathfrak{I}}\left\{{u}_{A}\left(t\right)\right\}$ by ±Δ*ν*. This results in an interference whose center is defined by the beat frequency, and with a bandwidth proportional to Alice’s bandwidth. This is an important point because it means that the overlap between the interference and Alice’s data cannot be avoided if the beat frequency is smaller than the signal bandwidth, i.e., if $\left|{\nu }_{A}\,-\,{\nu }_{M}\right| \, < \, B$.

This simple model of the SINR is used to obtain the semi-analytical BER shown in Fig. 5b. For simplicity, we assume that a single bit is transmitted in the form of a non-return-to-zero rectangular pulse of duration *T* related to the data rate *B* = 1/*T*. We then compute the SINR using Eq. 5 assuming a uniform spectral noise, and we deduce the BER from the fit shown in Fig. 4. The resulting calculated BER (blue curve in Fig. 5b) matches the experimental results very well. As can be seen, the degradation of the link is maximized when Mallory operates at the same carrier frequency as Alice ($|{\nu }_{A}\,-\,{\nu }_{M}|\,=\,0$), but also when the beat frequency is at half of the bandwidth of Alice’s transmission ($\left|{\nu }_{A}\,-\,{\nu }_{M}\right|\,=\,B/2$). This result suggests an optimization strategy for Mallory which avoids placing her jamming signal at the same carrier frequency as that used by Alice (thus minimizing the likelihood of detection). In general, as long as the interference signal is within Bob’s bandwidth, Mallory can have a disruptive effect on the Alice-Bob link. For larger detuning, when the interference signal exceeds this data bandwidth, the effect on the BER is negligible, as can be seen by the abrupt decrease in the modeled BER when the beat frequency increases. This result has profound implications, as it quantifies the extent to which the use of a broader transmission bandwidth (higher data rate) increases the vulnerability to jamming.

### Modulated jamming

**Fig. 6:**
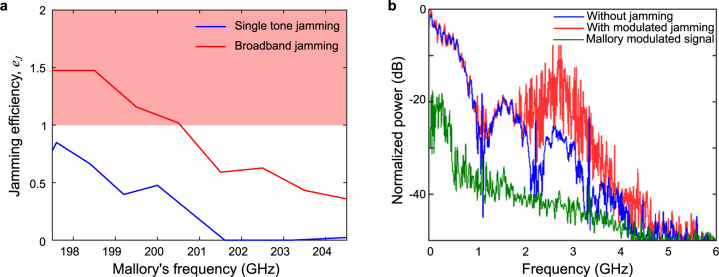
Modulated jamming. **a** Sweeping Mallory’s frequency from 197.5 to 205 GHz for the single-tone jammer (red line) and the modulated jammer (blue line), shows a difference in the jamming efficiency. The modulated jammer achieves jamming efficiency of 1 and completely destroy the signal (red region). **b** Bob’s received spectrum when jammed (red) shows an interference signal due to Mallory’s jamming attack with a modulated data (green), compared to Alice’s original signal (blue). Here, Mallory’s center frequency is at 200 GHz.

Finally, we consider the possibility that Mallory can use a modulated jamming signal rather than a single-frequency tone. To explore this idea, we equip Mallory with the possibility to transmit a random bit pattern (also OOK modulation) at a data rate of 0.5 Gbps. The results, presented in Fig. 6, show that this method of jamming is even more effective than the single-tone jamming scenario. With the same transmit power, Mal achieves ${e}_{J}\ge 1$ while operating with carrier frequencies between 197.52 and 200.5 GHz, completely destroying the communication link (Fig. 6a). Moreover, when Mallory’s frequency is greater than 201.5 GHz, the single-tone jamming is ineffective, yet the modulated jamming can still achieve a minimum *e*_*J*_ of 0.75. This phenomenon can be explained by reevaluating the analytical expression of Bob’s jammed signal. We introduce a new modulated signal term ${u}_{M}\left(t\right)$ in the second term of Eq. 1. The resulting expression, after eliminating the high frequency and DC terms, is:

7 $${\left|{E}_{B}(t)\right|}^{2}\,=\,\frac{{A}_{A}^{2}{{u}_{A}\left(t\right)}^{2}}{2}\,+\,{\frac{{A}_{M}^{2}{u}_{M}{\left(t\right)}^{2}}{2}} +{A}_{A}{A}_{M}{u}_{A}\left(t\right){u}_{M}(t)\, {{{{{\rm{cos }}}}}}\left(2\pi \Delta \nu t\,+\,\Delta \varphi \right)\,+\,N(t)$$

As earlier, this expression includes a sinusoidal interference term at the beat frequency Δ*ν*, but it is now also a function of Mallory’s data stream, *u*_*M*_(*t*). In addition, a new DC term that corresponds to Mallory’s modulated data (second term on the right-hand side of Eq. 7) is now part of the interference signal. Figure 6b shows the spectra that Bob measures without (blue) and with (red) Mallory’s modulated jamming, while the modulated data that Mallory sends is shown in green (measured while blocking Alice).

The DC term that Mallory introduces in the spectrum can have significant impact on the jamming. Importantly, this term allows Mallory’s carrier frequency to be very different from Alice’s and still maintain a successful jamming attack. For example, in our experiments, Bob’s zero-bias Schottky diode receiver is sensitive to a wide band of frequencies from 140 to 220 GHz. Therefore, as long as Mallory sends her modulated data in this band, the DC interference term can still disrupt the link. Then, by tailoring the bandwidth of her modulated signal, she can overlap Alice’s data to completely destroy it.

In conclusion, we report the first investigation of jamming at THz frequencies in a noncoherent modulation scheme. We demonstrate that Mallory, the jammer, can effectively interfere with Alice, the transmitter, by aiming at Bob, the receiver, and coupling into one of the side lobes of his antenna. This aiming requirement contrasts with jamming attacks at lower frequencies and finds its origin from the use of highly directional antennas at THz frequencies. Mallory’s attack can be optimized by adjusting her angular position and transmit power. In our testbed demonstration, we show that if Mallory increases Bob’s received power by only ~0.25 dB, she can disrupt the link by 50%, while she completely disrupts it with an increase of only ~0.75 dB. Interestingly, the eye diagrams reveal that Mallory’s jamming affects only the high bit, which means that Bob must adjust the value of his decision threshold to correctly decipher the transmission. More importantly, we emphasize the new ability for Mallory to tune her carrier frequency away from that used by Alice and Bob, which can minimize her likelihood of detection without substantial sacrifice in her ability to jam the channel, a method that we term “beat jamming.” Finally, we show that the attack can be even more devastating if Mallory uses a modulated signal.

One of the unique aspects of jamming at such high frequency is that even a single-tone source located far from Alice’s center frequency can disrupt Bob’s measured signal (assuming of course that Bob’s receiver is able to detect the signal) via convolution of Alice’s spectrum with Mallory’s single-tone. This jamming mechanism is possible because Bob uses an envelope power detector—in our case, a Schottky diode. This type of noncoherent detection is in common use at THz frequencies and is part of the recent standardization efforts of the IEEE 802.15.3d task group^20^. It is intended to be used for low-cost and low-complexity devices in short range applications. In this work, we used OOK modulation, and showed how the jamming affected the bits 0 and 1 differently. For more advanced modulation schemes, such as coherent modulation, multi-carrier transmission and high-order modulation formats, additional research would be required to evaluate the efficacy of these types of jamming attacks. In addition, we have made several simplifying assumptions about the threat model, for example that Mallory is a constant jammer, not broadcasting randomly or in reaction to the activity on the channel. These assumptions facilitate our focus on those aspects of jamming that are of particular relevance to the millimeter-wave and terahertz ranges and could be refined in future research.

As for Alice and Bob, they have several counter-measures available to them. If Alice increases her power or Alice and Bob increase their directivity gains, Mallory will either be less effective or would need to increase her transmit power or directivity to maintain the same level of attack. To detect the attack, Bob can monitor changes in his detected power and/or spectrum. He can also monitor the eye diagrams, as the jamming has a nonuniform effects on the bits 0 and 1. More cleverly, Alice and Bob can use different interference mitigation strategies^22,32–36^. For example, they can use a low-weight channel coding scheme^37^ where more information is conveyed through the bit 0, therefore reducing the disrupting effect of Mallory’s jamming. They can also use a binary pattern modulation^33^, a chirp spread spectrum technique^35^, or phase-domain spreading^36^, which have shown to be robust against interference. Of course, if Mallory takes a non-static approach to jamming (no longer a constant jammer), the situation becomes more complex, requiring further research. Our findings reveal important vulnerabilities which may redefine aspects of the physical layer security in the THz range.

## Methods

### Experimental setup

The detailed experimental setup is depicted in Fig. 1b. Alice’s transmitter consists of a photoconductive antenna (PCA) illuminated by two slightly detuned 1535 nm distributed feedback (DFB) diode lasers. The difference in frequency between the two lasers is used to generate THz radiation through a photomixing process in the PCA^38^. For wireless communications, we modulate the optical signal using a fiber-coupled lithium niobate Mach-Zender modulator driven by a pulse pattern generator (PPG) outputting a 1.12 Gbps OOK signal with a pseudo-random binary sequence of length 2^7^ − 1^39^. Before reaching the photomixer, the optical signal is amplified with an erbium-doped fiber amplifier (EDFA). Mallory’s antenna consists of a frequency multiplier chain, with a multiplication factor of 16, driven by an RF oscillator (RF OSC) outputting 12.2–12.8 GHz. For broadband jamming, the driving RF signal is modulated using a double balanced mixer (DBL Mixer) with a limited bandwidth of 500 MHz driven by a PPG^40^. Bob’s receiver is a waveguide-coupled zero-bias Schottky diode with a detection sensitivity limited by the bandwidth of the waveguide, 140–220 GHz. The received signal passes through low-pass filters to obtain the baseband signal between 0.1 MHz and 6 GHz. The signal can then be routed to a power meter, an oscilloscope, or a real-time bit-error rate tester (BERT), which allows us to measure the bit error without any additional post-processing.

Alice’s antenna is positioned at 210 mm from Bob’s antenna, while Mallory is 280 mm away from Bob. Alice uses a 75 and 120 mm lens to focus her beam into Bob’s antenna while Mallory only uses a 75 mm lens. Mallory and Bob both use WR5.1 conical horn antennas. Mallory’s rotation angle can be adjusted from 20° to 45° relative to the Alice to Bob line-of-sight, limited at the minimum by the physical size of Alice’s lenses. This attack can be effective at lower angles given that Alice and Mallory do not physically block the beam of each other.

### Effectiveness of jamming based on Friis equation

Mallory’s jamming depends on how well she can couple into Bob’s antenna relative to Alice’s coupling. This can be evaluated using the Friis transmission equation. Mallory’s power detected by Bob is:

8 $${P}_{{{\rm{Bob}}}}^{{{\rm{Mallory}}}}\,=\,{P}_{{{\rm{Mallory}}}}{G}_{{{\rm{Mallory}}}}^{\theta \,=\,0}{G}_{{{\rm{Bob}}}}^{\theta \,=\,{\theta }_{M}}{\left(\frac{\lambda }{4\pi {R}_{{MB}}}\right)}^{2}$$

where *P*_*Mal*_ is Mallory’s power, *λ* is the wavelength and *R*_*MB*_ is the distance between from Mallory to Bob. ${G}_{{Mallory}}^{\theta \,=\,0}$ is the maximal gain of Mallory’s antenna (in the front direction, *θ* = 0, if she uses, e.g., a horn antenna). ${G}_{{Bob}}^{\theta \,=\,{\theta }_{M}}$ is the gain of Bob’s antenna in the direction from Mallory to Bob, *θ* = *θ*_*M*_. Similarly, the power from Alice that Bob detects is:

9 $${P}_{{{\rm{Bob}}}}^{{{\rm{Alice}}}}\,=\,{P}_{{{\rm{Alice}}}}{G}_{{{\rm{Alice}}}}^{\theta \,=\,0}{G}_{{{\rm{Bob}}}}^{\theta \,=\,0}{\left(\frac{\lambda }{4\pi {R}_{{AB}}}\right)}^{2}$$

where, we again assume that the maximal possible gains are taken in the forward directions, *θ* = 0. Then, the effectiveness of Mallory’s jamming can be evaluated by computing the ratio of powers of Mallory and Alice as measured by Bob’s receiver, which can be found in Eq. 4.

The exponential fit in Fig. 3b is represented by the following expression: ${e}_{j}\,=\,a\,{{{{{\rm{exp }}}}}}\left(-b\Delta {{{\rm{Power}}}}_{{{\rm{Bob}}}}\right)\,+\,c$. Using a least-square fitting procedure, we get *a* = −1.747, *b* = 0.9735 and *c* = 1.885. This fit describes the change in power detected by Bob’s receiver needed to in order to induce a change in jamming efficiency.

### Error probability fit of the SINR

The fit in Fig. 4 is calculated from the analytical model of the probability of error for a noncoherent system with OOK modulation, which can be derived from a Rayleigh and Rician probability distribution functions used to model the envelop statistics, and which can be subsequently used to evaluate the probability of symbol error^30^. We use the following modified theoretical function to fit our measurements:

10 $${BER}\left({\gamma }_{b}\right)\,=\,\frac{1}{2}\left[{e}^{-\frac{{\gamma }_{b}}{2}\,+\,B}\,+\,\frac{1}{2}{{{{{\rm{erfc}}}}}}\left(\sqrt{\frac{{\gamma }_{b}}{2}}\,-\,C\right)\right]$$

where ${{{{{\rm{erfc}}}}}}\left({{{{{\rm{x}}}}}}\right)$ is the complementary error function. The fitting parameters *A*, *B*, and *C* were obtained by minimization of the mean-square between the analytical model and the experimental results. The energy per bit ${\gamma }_{b}\,=\,{{{{{\rm{SINR}}}}}}\,\cdot\, A$ is proportional to the SINR through the parameter *A*, that we fit to obtain *A* = 7.77. In general, this parameter depends in a complicated manner on the transmission parameters (free-space path loss, antenna gains, distances, etc.), but also on the bandwidth, spectral efficiency and modulation scheme. The parameters *B* and *C* are used to consider the fact that the decision threshold for each BER measurement is not set at the standard half point but is rather optimized for each measurement to reduce the effect of jamming on the upper bit and minimize the BER. In Fig. 4, the slight deviation between the model (black curve) and the points for the case of optimized threshold (yellow to blue points) finds its experimental origin in the limited voltage resolution of the decision threshold in the BERT.

### Effect of the jamming on the channel capacity

Inset of Fig. 4 shows the normalized channel capacity $\widetilde{C}$ as a function of the interference term defined as:

11 $$\widetilde{C}\,=\,\frac{{{{{{{\rm{log }}}}}}}_{2}\left(1\,+\,{{{{{\rm{SIN}}}}}}{{{{{{\rm{R}}}}}}}_{{{{{{\rm{Jammed}}}}}}}\right)}{{{{{{{\rm{log }}}}}}}_{2}(1\,+\,{{{{{\rm{SN}}}}}}{{{{{{\rm{R}}}}}}}_{{{{{{\rm{Unjammed}}}}}}})}$$

Here, ${{{{{{\rm{SINR}}}}}}}_{{{{{{\rm{Jammed}}}}}}}\,=\,S/(I\,+\,N)$ is the measured SINR in the presence of Mallory’s jamming signal, while *S* is Alice’s signal, *I* is the interference caused by Mallory, and *N* is the noise. ${{{{{{\rm{SNR}}}}}}}_{{{{{{\rm{Unjammed}}}}}}}\,=\,S/N$ is the SNR that Bob detects in the absence of a jammer (*I* = 0), which is experimentally measured without Mallory’s presence. With this normalized definition, $\widetilde{C}\,=\,1$ indicates no effect of the interference and the channel capacity is unaffected, while lower values indicate how much of the channel capacity has been negatively impacted by Mallory’s jamming. For example, when $\widetilde{C}\,=\,0.4$, the channel capacity decreased to 40% of its original value.

### Reporting summary

Further information on research design is available in the Nature Research Reporting Summary linked to this article.

## Supplementary information

Reporting Summary

## Data Availability

The data that support the findings of this study are available from the corresponding author upon reasonable request.
